# Digitalisation before and after the Covid-19 crisis

**DOI:** 10.1007/s12027-021-00656-8

**Published:** 2021-02-25

**Authors:** Kai Härmand

**Affiliations:** Judge at Harju County Court, Kummeli tee 46, 11912 Tallinn, Estonia

**Keywords:** Digital company law, Remote annual meeting, Digital authentication

## Abstract

States all over the world have quickly amended legislation in order to help businesses conduct their activities remotely and online. In this article, we will see examples of existing rules concerning annual general meetings being better implemented (in Italy), of legislation being amended with temporary rules (in Germany) and of political momentum being used to bring about fundamental changes (in Estonia).This article provides a brief overview of how changes have been made in order to allow virtual annual general meetings in different countries and what kind of changes were made, and also provides a somewhat deeper look at Estonia’s new legislation concerning remote notarial transactions, online annual meetings and digital infrastructure.

## Introduction

Digital technologies are changing the face of public administration and the economic landscape, as well as the way we do business and communicate. They allow more tailor-made, proactive public services and permit economic players to provide more innovative solutions. Technological change will work only with the cooperation of businesses, Member States, regions and European Union (EU) institutions. The Covid-19 pandemic has given a unique possibility to take a huge step forward in digitalisation of corporate governance and the company law area. To jump to our conclusions, the most crucial challenge is to make sure that the benefits of digitalisation are not hampered by the traditional approach taken in other areas of law. This article focuses on legislation that allows businesses to hold their annual general meeting (AGM) virtually, and provides an overview of the legislation before the Covid-19 crisis, as well as emergency regulations that Member States adopted during the crisis, and charts the way forward.

## Legal framework before Covid-19

The annual general meeting (AGM) is one of the core instruments for executing shareholders rights and therefore the legal regulation concerning the holding of one is rather detailed^1^ at EU level and also in Member States. For some years,^2^ in the United Kingdom^3^ and in most states of the United States, it has been possible for companies to hold a virtual-only AGM, but practice has varied strongly. One of the key questions has been how to interpret legislation referring to the “place” of a meeting – meaning a physical place.

The common understanding of a virtual annual general meeting means conducting the meeting online, and allowing all members or shareholders to attend virtually. Attendees of a virtual AGM should be allowed to interact, ask questions, vote and participate in real time, as they would do at a physical meeting. A hybrid AGM is a mixture of virtual and physical meetings, where members can decide to meet either online or physically. This removes the need for members or shareholders to be in one location, and ensures higher attendance and engagement. This is important not only for listed companies with shareholders living all over the world but also for small and medium-sized enterprises (SMEs), not-for-profit-organisations and multinational start-up companies.

### Virtual annual general meetings and corporate governance before Covid-19

#### Estonia

Under pre-Covid crisis legislation, the possibility of using electronic means for holding annual general meetings and taking decisions varied greatly from one legal entity to another. To a certain extent, private limited companies and public limited companies have similar opportunities. In private limited companies as well as public limited companies, the articles of association could provide for the possibility to vote on draft resolutions prepared on agenda items by electronic means before or during the meeting.^4^

For non-profit associations, for-profit associations, foundations and apartment associations, this possibility was almost non-existent. The adoption of decisions by means of a statement of intent in writing or in a form that could be reproduced in writing without convening a meeting was possible for apartment associations^5^ and for private limited companies. In this case, there was no meeting as such, but only a vote on the draft decisions. For non-profit associations, written voting was possible if all members voted in favour of a decision. That made it difficult to use this option in practice. For commercial associations^6^ written voting was possible in case certain conditions were met (e.g. the association has to have more than 200 members).

#### Italy

Italian law has allowed shareholder meetings to be conducted and attended online for several years now. The relevant provisions are in the Civil Code.^7^ Up to now, however, only a limited number of companies have used that opportunity because in implementing the legal regime,^8^ the practice has been that, in order for a meeting to be validly held, at least two persons must be physically present in the same place.

#### Poland

In Poland, the possibility of a remote shareholders meeting is one which has for long been provided for in legislation. The amendment to the Commercial Companies Code^9^ from August 2009 stipulated that companies listed on the Warsaw Stock Exchange have an obligation to offer the possibility of online participation in annual general meetings. This requires a real-time broadcast of the meeting and two-way communication of participants at it and most of all the possibility of exercising voting rights in person or by proxy before or during the general meeting. Provisions of the Commercial Companies Code regulating shareholders meetings were again changed in 2019 and on 3 September 2019 the consequent amendments came into force. The amended legislation permitted limited liability companies and joint-stock companies to hold shareholders’ meetings by means of electronic communication in the event that this was explicitly permitted in the company’s articles of association.

In a nutshell, the Polish regulation before the Covid-19 crisis of the annual general meeting stipulated as a general rule, that an AGM be held as a physical meeting.^10^ As an exception^11^ to that rule, decisions of the shareholders of a limited-liability company and joint-stock companies could be adopted without holding a meeting if all shareholders consented to the given resolution in written balloting. The law stipulated an opt-out possibility. In case shareholders did not wish to have the option of holding such meetings, they had to amend their company’s articles of association accordingly.

Remote participation included in particular^12^ real-time two-way communication by all persons participating in the shareholders’ meeting, with the possibility of speaking during the meeting; and also exercising voting rights during the shareholders’ meeting both personally or by proxy. A company was required to provide confirmation that a vote had been submitted and a person was entitled to request confirmation that his or her votes had been properly recorded and counted.

The detailed technical requirements of the virtual participation had to be specified in bye-laws adopted by the supervisory board in the case of a joint-stock company or by the shareholders in the case of a limited liability company. Such bye-laws could be adopted by a shareholders’ resolution without holding a shareholders’ meeting if shareholders representing an absolute majority of votes agreed in writing to do so. Technical requirements were also required to describe how the identification of persons will be conducted and the way in which a proxy was obliged to provide confirmation of his or her authorisation to represent the shareholder.

## Covid-19 emergency legislation in some EU countries

In response to the spread of Covid-19 and the lock-downs introduced in response, many countries updated their regulations with temporary provisions allowing companies to hold virtual and hybrid AGMs in order to encourage the use of remote attendance and remote voting mechanisms by shareholders.

### The Polish approach

Although Polish law already contained provisions for remote shareholders meetings, a new Covid-19 law was adopted. The amendment^13^ came into force on 31 March 2020 and abolished all impediments to conducting AGMs using electronic means of communication, even if the statute of a company did not include this option. The possibility of participation in a meeting by means of electronic communication is decided upon by the person convening that meeting, and in the case of a limited liability company, information on convening a meeting should include information on how to participate in the meeting, speak during it, exercise voting rights and raise objections. As has already been noted, the possibility of participation by means of electronic communication is decided by the person convening that meeting: the information on convening the meeting must include the information on how to participate, how to exercise voting rights and how to raise objections. There are however some restrictions. The meeting must be held at the place where the company is registered and in the territory of Poland. The chairman of the meeting and the secretary/notary public must be physically present. Ordinary and extraordinary shareholders’ meetings, board meetings and other forms of meetings can be held virtually.

Similar obligations regarding the information to be included in the invitation are imposed on listed companies, – except for the instructions on how to object to resolutions adopted at the meeting.

In addition to the changes in company law, the deadlines for approval of annual financial statements and annual consolidated financial statements and the annual shareholders’ meetings have been extended by the Regulation by the Minister of Finance dated 31 March 2020. The extension for limited liability companies and joint stock companies was three months and for listed companies was two months.

### The German approach

Although Art 118(1)(2) of the Stock Corporation Act lays down regulations for electronic meetings if these are provided for either in the articles of association or by the management board, shareholders may, attend a meeting without being physically present and exercise their rights by electronic means. It has been explained in the legal literature that the reform carried out in 2002 is explicit in that the legislator accepted participation in the general meeting by electronic means.^14^ Germany made changes to the German Stock Corporation Act^15^ from 28 March 2020 and stipulated the clear possibility of holding annual general meetings only virtually. The criteria for the virtual meeting are that the entire meeting should be broadcast with the opportunity of asking questions (electronically or otherwise), and voting has to be conducted and authorised digitally (or alternatively by post). The convening period has been reduced from 30 to 21 days. The time limit for the AGM has been extended from eight months to a fiscal year.

### The Austrian approach

According to Art 104 of the Austrian Stock Corporation Act, annual general meetings of shareholders in Austria must be held physically. As an emergency measure during the Covid-19 pandemic, amendments were made to legislation.^16^ The company law-related amendments were introduced in the Second Covid-19 Act and extended in the Fourth Covid-19 Act. According to Art 1(1) of the Fourth Covid-19 Act for the purpose of preventing the spread of Covid-19, meetings of shareholders and meetings of company officers of a corporation, a partnership, a cooperative, a private foundation or an association, a mutual insurance foundation, a small insurance foundation or a savings bank may also be held without the physical presence of the participants and resolutions may also be passed in a different manner, subject to statutory orders. The new regime for annual general meetings is to be permitted until the end of 2020. More detailed regulations for corporations and other company forms have been reserved for a statutory order issued by the Federal Minister of Justice. The eight-month convocation period of the annual general meeting of a corporation’s shareholders has been extended to twelve months.^17^ The Fourth Covid-19 Act also provides the same opportunity for limited liability companies.^18^

The legislator also extended the time limits for submitting annual fiscal statements^19^ starting from 22 March 2020 and ending on 30 April 2020, with the possibility of this time limit being further extended by a statutory order issued by the Federal Minister of Justice – this extention was made with the Fourth Covid-19 Act.

### The Italian approach

On 8 and 9 March 2020, the Italian government issued two Presidential Decrees imposing temporary restrictions on movement and on assembly in order to curb the spread of the Covid-19 virus. Obviously, these restrictions were an impediment to annual general meetings as well. On 17 March 2020, the Italian government issued an emergency law-decree, Law Decree No. 18 (“Cura Italia Decree”),^20^ allowing all listed Italian legal persons to hold their AGMs remotely either entirely or in part, or by designating a proxy, even if this is not permitted by their bye-laws.

The amendment^21^ specified that companies could use the right to make announcements in the notice of a shareholders’ meeting to stipulate that participation would take place exclusively through the representative appointed pursuant to Art. 135 of Legislative Decree no. 58/98. Paragraph 2 of Article 106 of the same Decree stated that a company has to guarantee the identification of persons who attend the shareholders meeting, their participation and the exercise of their voting rights.

In the light of the circumstances surrounding Covid-19, Italy changed the provisions regarding remote participation in annual general meetings, and the Council of Public Notaries of Milan issued recommendation no. 188^22^ on the implementation of the legal regime, explaining that the statutory clauses do not prevent shareholders’ meetings from being held with the remote participation of all attendees. The Recommendation also specifies that, in such circumstances, all members of administrative and control bodies, the designated representative, the secretary or the notary public, as well as other persons who are allowed to participate in the shareholders’ meeting in accordance with the law, the bye-laws and the shareholders’ meeting regulations, can attend a shareholders’ meeting by means of telecommunications, but not the shareholders, who must necessarily attend the shareholders’ meeting exclusively through the designated representative.

### Questions arising due to emergency regulation

A number of EU Member States have adopted temporary or emergency company law legislation, among them the Netherlands, Belgium and France. As Sweden imposed no restrictions on movement, no new legislation was adopted by it regarding virtual annual meetings.

New provisions have certainly brought flexibility for shareholders by making virtual attendance at shareholders’ meetings permissible. Such flexibility is particularly convenient for foreign shareholders because it frees them from the effects of any restrictions on movement or other difficulties. Some questions still remain however, especially regarding the temporary nature of such regulation and technical irregularities.

The identification of participants is the first obvious problem and the stability of technical equipment the second one. Even the best internet connection may fail and shareholders need to have certainty that their voting rights have been validly exercised. The issue is relevant in many ways starting from liability issues and ending with possibilities to collect and present evidence in court.

## The Estonian example and response to Covid-19

To explain and understand the Estonian response to the Covid-19 outbreak, one needs to go back and explain the infrastructure of its digital governance in general.

### Estonian digital infrastructure

#### ID-card and x-tee: the core of e-governance in Estonia

The backbone of e-governance in Estonia is *x-tee*^23^ which is a technological internet-based highway, the data exchange layer for different information systems for secure data exchange.^24^ X-tee enables information systems to communicate with each other and exchange relevant data. A member of X-tee describes the shared data and other members can use that data based on an agreement. Due to the large number of public and private systems that have joined X-tee, all members of X-tee can use the services and data of other members to improve their own business processes. X-tee has a versatile security solution: authentication, multi-level authorisation, a high-level system for processing logs, and data traffic that is encrypted and signed.

Over 1000 public and private sector organisations and enterprises in Estonia use X-tee daily and more than 52 000 different users benefit from the system.^25^ It is said that it saves 844 years of working time every year. Today, it is implemented in Finland, Kyrgyzstan, the Faroe Islands, Iceland, Japan and other countries. Similar technology based on the Estonian interoperability experiences has also been implemented in Ukraine and Namibia. Members of federated ecosystems can publish and use services with each other as if they were members of the same ecosystem. Federation enables easy and secure cross-border data exchange between these ecosystems. A federation between Estonia and Finland was established in February 2018.

Every resident, every physical person in Estonia has a unique identification code that enables a person to use a secure authentication and electronic signature using ID-card, Mobile ID or Smart ID solutions.^26^ X-tee and secure authentication solutions enable the implementation of the “once only” principle, that is an e-government concept that aims to ensure that citizens, institutions, and companies only have to provide certain standard information to the authorities and administrations once.

#### Estonian e-Business Register: History at glance

The e-Business Register is a service based on the database of the registry department of the county court and displays the real-time data of all legal persons registered in Estonia.^27^

The first IT-system for the Estonian Business Register was created in 1995 at the same time our Commercial Code^28^ came into force and in 2002 electronic register gets legally recognized by the law. A big step forward was taken in 2005 when electronic submission of applications by citizens and entrepreneurs was allowed.

The Company Registration Portal, an e-Business Register as it is known today– with one window for each citizen – was created in 2007 and since then, several services have been added. It is worth mentioning that companies may file their annual report electronic (the XBRL) since 2010. An accounting system – e-Financials for SMEs – was created in 2014.

Today the Company Registration Portal^29^ is a public service environment which allows the submission of documents to the Business Register electronically, without the need to use a notary’s services. It can be used to establish new businesses and non-profit organisations or to submit any kind of applications to a court. It is possible to amend, liquidate or delete registry data. Annual reports can also be compiled and submitted entirely electronically. The e-reporting environment allows the preparation of the main reports and the notes, adding notes and documents, digitally signing the annual report or printing and signing it on paper, and also allows the sworn auditor to prepare the sworn auditor’s conclusions and to certify them electronically.

At present it is possible to enter the portal with an Estonian, Latvian, Finnish or Belgian ID card or an Estonian or Lithuanian Mobile ID.

From 2011, most companies have been established over the internet using the e-Business Register and the average speed of the registration process has come down from five days to a couple of hours. Today 98% of companies are established online and the e-Business registry record speed for establishing a company is sixteen minutes.

The e-Residency^30^ programme also allows non-Estonian citizens to access the register and use digital solutions when establishing a company in Estonia. The e-Business register makes the process of registering a company and submitting documents like annual reports easy and efficient for users online no matter where they are.

The e-Business Register makes the life of the entrepreneur much easier and reduces the administrative burden. A business has a single unified reporting environment which makes it convenient to enter and submit data, because there is one certain place, format and way of submitting all required data. Electronic submission simplifies the organisation of reporting and reduces duplication in submitting data.

### The Estonian response to the Covid-crisis

Retrospectively one may say that preparations for the lock-down started in Estonia already in 30 January 2019 when the Estonian Parliament, the *Riigikogu* adopted amendments to the Notarisation Act^31^ that introduced remote authentication of some transactions. The amendments made it easier for e-residents and also Estonian citizens to perform transactions and operations that require a notarial form abroad. The amendments created a new form of remote notarial certification, which enables the certification of some transactions *via* video link. For example, transactions and proxies for the transfer and pledge of a private limited company’s shares, as well as the submission of marriage and divorce applications and applications for relating to the inheritance of property or the waiver of an inheritance. At first performing remote verification operations was possible only in an Estonian embassy or other type of foreign mission. The Notarisation Act was amended once more shortly before the emergency situation was announced^32^ so as to allow almost all transactions that needed notarial certification to be conducted remotely. In May, the *Riigikogu* amended the Commercial Code with provisions that allowed all legal persons to conduct annual general meetings (or any other meetings of other legal bodies of the legal person) virtually.

#### Remote authentication of notarial acts^33^

Remote authentication enables the conduct of notarial acts *via* a video link created between the notary and the customer. Such authentication is equivalent to authentication at a notary’s office. The law clearly states that remote authentication is an opportunity, not an obligation. The decision to allow remote authentication is made by the notary. Remote authentication may be carried out either at a place suited to the customer or at an Estonian embassy.^34^ Transactions using remote authentication can be conducted in five Estonian embassies – Helsinki, Stockholm, Brussels, Riga and London. It is possible to carry out the required act *via* the self-service portal of the Chamber of Notaries for Estonian citizens with digital ID or an e-Resident’s digital ID. The self-service portal proposes available times that suit both the notary and the embassy. The portal displays relevant documents 24 hours before the scheduled beginning of the transaction and the user can examine the transactions details. Estonian notaries use the Veriff Facial Recognition Programme to identify parties conducting notarial acts using remote authentication. The service is provided centrally through the Chambers of Notaries. The Veriff web page is displayed in the language that the customer has used in the self-service portal, but the language can be changed, if need be. The transaction takes place in the notarial self-service portal.

The notarisation process itself as conducted *via* video link does not differ from the process in the notary’s office. In order to examine the data of the act, the notary displays the document on the screen, which can then be studied. After the document has been examined, the notary makes it available for the parties so that they can digitally sign it.

Statistics shows that remote transactions are a growing trend and that the total number of notarised transactions has not decreased during the crisis. In fact, it has slightly increased due to apostilles and other verification transactions needed for transactions conducted abroad. In October, notaries performed 692 notarial acts, then already in November, 1035. Through the e-service launched at the beginning of February this year, more than 7,000 acts have already been performed without a person having to physically visit a notary office. In November, notaries performed a total of 28,967 official acts, (in comparison, there were 6900 real estate transactions).

Remote authentication is used also in transactions in the field of company law in the event that the owner or the representatives of a company (or any other legal person) do not have access to Estonian digital services with an Estonian digital ID or e-residency card.

#### Extension of electronic possibilities for organizing meetings and adopting decisions

In 20 April 2020 the Estonian Parliament initiated amendments to the General Part of the Civil Code Act and other Acts to extend the decision-making capacity of all legal entity bodies without the need for a regular meeting requiring physical presence. Under current law, the capacity to use electronic means for holding meetings and taking decisions varies greatly from one legal entity to another. The purpose of the amendment was to enable participation in the meetings of all legal persons by electronic means and to allow the alternative of taking decisions in writing without holding a meeting. The draft removed the requirement that the list of participants in the general meeting that is annexed to the minutes of a general meeting of members of a non-profit association must be signed by hand. According to the previous regulation, each of the participants in the general meeting of a non-profit organisation had to sign the list of participants as an annex to the minutes, the original of which was submitted (sent by post) even if the entry application was submitted electronically. The amendment harmonised the requirements for the registration of participants and the preparation of minutes of the general meeting of members of a non-profit association with the requirements applicable to other associations. In addition, the draft also contained an amendment to the Commercial Code Amendment Act (transfer of shares), which gave effect to the abolition of the formal requirement for the transfer and pledge of shares as soon as possible. Enforcement of the abolition of the formal requirement for transfer and pledge of shares was necessary because notarisation of transactions has become difficult in crisis situations due to restrictions on movement imposed by states, as well as due to the fact that issuing and sending foreign documents is at present taking more time than usual.

It is also important to note that, although the need for amending the legislation was particularly clear during the state of emergency declared on 12 March 2020, the solutions proposed in the draft were intended to be permanent and to be used after the end of the state of emergency. The amendments came into force^35^ on 24 May 2020, a month after the initiation of the draft in the Parliament.

## Conclusion

States all over the world have quickly amended legislation in order to help businesses conduct their activities remotely and online. We have seen good examples of existing rules concerning annual general meetings being better implemented (in Italy), of legislation being amended with temporary rules (in Germany) and of political momentum being used to bring about fundamental changes (in Estonia).

Regarding virtual shareholders’ meetings and doing business online, there are legal questions, such as who bears the burden of liability for inadequate technical measures used for the meeting and whether shareholders are being deprived of certain rights. Unified authentication opportunities create the possibility of developing a secure online platform for conducting meetings.

How can transparency be assured during a pandemic when board meetings and annual general meetings are not physically held and are held behind closed doors? Should governments continue to allow emergency solutions only or is this a time for a digital turn in company law?

All EU documents that set the vision of how we do business in the future, namely the small and medium enterprise (SME) strategy for a sustainable and digital Europe, mention technological change and digitalisation. The strategic aim is to reduce the regulatory and administrative burden, but the approach – one in, one out principle- is too “mathematical”. One should ask whether the obligation is necessary at all and how it can be automated. The data that is already in registries and databases governed by the state should be used, with the consent of a business of course, in the benefit of a company, for example annual reports, at least part of it, can be formed automatically, because the data is already reported and collected by tax office.

Authentication and identification have to work cross-border and it is not a member state governance issue to have or not to have digital signatures and means of identification. It is an EU issue and should be solved at EU level. If the state (or Member States together) is not doing it, the private sector will do it (or has done it already), but in that case it may be fragmented which gives some big corporations significant power over other businesses.

## Footnotes

1 Directive 2007/36/EC of the European Parliament and of the Council of 11 July 2007 on the exercise of certain rights of shareholders in listed companies *OJ L 184, 14.7.2007, p. 17–24* https://eur-lex.europa.eu/legal-content/EN/TXT/?uri=celex%3A32007L0036 and Directive (EU) 2017/1132 of the European Parliament and of the Council of 14 June 2017 relating to certain aspects of company law OJ L 169 30.06.2017 p 46-127 https://eur-lex.europa.eu/legal-content/ET/TXT/?uri=CELEX%3A32017L1132 [8, 9].

2 In June 2016, the EQS Group supported the first virtual-only AGM for Jimmy Choo in UK.

3 Companies Act 2006, art 360A https://www.legislation.gov.uk/ukpga/2006/46/contents [4].

4 Company Law Act art 170 (1) 1); art 298 (1) 1) RT I 1995, 26, 355 https://www.riigiteataja.ee/en/eli/523072020002/consolide [6].

5 Apartment Ownership and Apartment Associations Act, art 21, RT I, 13.03.2014, 3 https://www.riigiteataja.ee/en/eli/526102020001/consolide [1].

6 Commercial Associations Act, art 53, RT I 2002, 3, 6https://www.riigiteataja.ee/en/eli/526052020001/consolide [5].

7 Civil Code art 2370 (4); art 2479-bis (4) and art 2538 (6).

8 Legislative Decree no. 58/98, art 135 [13].

9 Polish Commercial Companies Code, Dz.U.2000. 94.1037, https://dziennikustaw.gov.pl/DU/2000/s/94/1037, consolidated text Dz.U.2013.1030 https://dziennikustaw.gov.pl/DU/2013/1030 [7].

10 Polish Commercial Companies Code, art 227 (1) [7].

11 Polish Commercial Companies Code, art 227 (2) [7].

12 Polish Commercial Companies Code art 234 §1 and art 406 §5 [7].

13 Act of 31.03. 2020 amending the Act on special arrangements for preventing and combating COVID-19, other infectious diseases and the crisis situations caused by them and certain other acts.

14 Kubis. MüKo zum AktG. 3. Auflage 2013. §118, Rn 15 [12].

15 German Stock Corporation Act, BGBl. I 1965, S. 1089; 2017, S 2446 https://www.gesetze-im-internet.de/aktg/ [2].

16 *Gesellschaftsrechtliches COVID-19-Gesetz*, COVID-19-GesG BGBl. I Nr. 16/2020 https://www.ris.bka.gv.at/Ergebnis.wxe?Suchworte=Gesellschaftsrechtliches+COVID-19-Gesetz%2C+COVID-19-GesG&x=0&y=0&Abfrage=Gesamtabfrage [10].

17 Fourth Covid-19-GesG art 2 (1) [10].

18 Fourth Covid-19 GesG art 2 (3) [10].

19 First Covid-19 Act, art 2 [10].

20 The Decree was published in the Official Gazette on the same day, at which time it became effective .

21 Legislative Decree no. 18, Art. 106, paragraph 2, unofficial English translation https://www.confindustria.it/wcm/connect/e633da49-7ac9-427d-bad0-23f9a78cd546/Decree-Law_18_17032020.pdf?MOD=AJPERES&CONVERT_TO=url&CACHEID=ROOTWORKSPACE-e633da49-7ac9-427d-bad0-23f9a78cd546-n4IhvQj [13].

22 This was announced by the Council of Public Notaries of Milan on 24.03.2020.

23 Estonian word “tee” means road in English.

24 Additional information can be found here https://www.ria.ee/en/state-information-system/x-tee.html.

25 Additional information can be found here https://www.x-tee.ee/factsheets/EE/#eng.

26 Additional information can be found here https://www.id.ee/en/id-card/.

27 https://www.rik.ee/en/e-business-register.

28 Commercial Code RT I 1995, 26, 355 https://www.riigiteataja.ee/en/eli/523072020002/consolide [6].

29 https://www.rik.ee/en/company-registration-portal.

30 Additional information can be found here: https://e-resident.gov.ee/.

31 Notarisation Act RT I 2001, 93, 564 https://www.riigiteataja.ee/en/eli/527122019008/consolide; *amendments* RT I, 22.02.2019, 2 https://www.riigiteataja.ee/akt/122022019002 [14].

32 An emergency situation was announced on 12.03.2020 in Estonia.

33 Additional information here https://www.notar.ee/en/teabekeskus/kaugtoestus

34 There are restrictions on remote authentication. Hence it can be used to perform all notarial acts – except certification of contracting marriage and divorce.

35 General Part of the Civil Code Act, RT I 2002, 35, 216, https://www.riigiteataja.ee/akt/123052020002 [15].
